# Indents

**Published:** 1919-12

**Authors:** 


### INDENTS

**☞Brief Articles of Technical or Scientific Value will receive notice and comment. Contributions invited.**

CHINOSOL IN ROOT FILLING. Root-canal fillings which are to be radiographed will have a denser outline if chinosol is incorporated with the chlora-percha, which should be pumped into the canal before inserting the gutta-percha point. Chinosol is a good antiseptic and is indicated for canals that have been putrescent.—Victor H. Fuqua, in *Cosmos.*

THAT PRECIOUS AMERICAN SOAP. You pay thirty-five cents for an ordinary piece of coarse, brown laundry soap in Prague, Bohemia, today, and toilet soap costs six times as much. Some soap has been sent in by the American Red Cross, but sending in soap enough to wash a nation is a big job, and this new republic of Czecho-Slovakia has few animals left from which to obtain soap fats. Neither does it have trees that produce vegetable oils.

In Rumania, too, soap is more to be desired than rubies or fine gold. A Red Cross worker, inspecting the hospitals in Bucharest, was met everywhere with accounts of the cures effected through the use of American soap. Two thousand sand cakes had been distributed at a military hospital at Oradea Mare and the doctors in charge declared that 500 cases of sore eyes had been cured principally through the use of this soap. At Bekes Csaba, soap again was praised as a remedy which had abolished skin diseases among hundreds of patients.

DESENSITIZING CERVICAL MARGINS. If you have never tried Buckley's desensitizing paste for this purpose, smear a little of the paste on the sensitive surface and cover it with a thin mix of calxine for two days and you will get satisfactory results.—E. G. Simpson, in *Digest.*

---

THE FUTURE OF DENTAL PRACTICE. To make up for the shortage of dental graduates the future dental practice must attach a *Dental Hygienist* as well as a laboratory *Technician*, and a competent office maid.

---

ETCHING THE GOLD INLAY. Cover the surface not to be etched with wax so as to expose only the surface to be etched. Moisten the exposed gold surface with seventy-five per cent. hydrochloric acid and then treat it with mercury. When the desired etching is obtained, heat the inlay in an open gas flame to evaporate the mercury and plunge into the usual solution. Wash and dry and a fine crystalline surface will appear which will securely hold the cement attachment.—F. W. Frahm, in *Pacific Gazette.*

---

TO FACILITATE GRINDING TOOTH SUBSTANCES. Make a paste of fine carborundum with glycerine and use it on the stones or disks used in cutting down tooth tissues. Care should be taken to keep the paste from working its way into the handpiece of the engine. The paste facilitates the cutting and relieves the pain considerably; it also prolongs the life of the disks and the tooth is readily cleaned of the debris.—R. L. Newling, in *Pacific Gazette.*

---

EXTRACTING CAUTION. A word of caution should be heeded in carrying out the demand for radical extractions. Teeth that can not be demonstrated infectious and that may be serviceable in assisting in the retention of partial dentures should not be extracted for fear that they may become infectious in the future. Many times a few teeth left in the jaws will help the patient to learn to wear dentures with less trouble than would be experienced if the mouth be made edentulous. If the patient learns to wear a partial plate and in the future any tooth appears to be a possible menace, it can be extracted and an artificial tooth can be added to the denture with little inconvenience to the patient. If we use a little judgment in our extracting our patients will not feel so badly about loosing their teeth. This applies, of course, to teeth that are not infected or that in all probability may not become a source of focal infection in the near future.

---

Pathology and Prophylaxis of Pyorrhea. Dr. Maurice Roy summarizes a long paper on the above subject presented to the Sixth International Dental Congress as follows:

1. When fully established pyorrhea is a disease characterized by the presence of a gingival pocket more or less deep at the neck of the tooth, with corresponding denuding of the root wherever it is in relation with the pocket.

2. Peri-cemental abscesses on living teeth are not due to gouty tophi; they are pyorrhea abscesses formed at the end of a winding pocket the opening of which at the neck of the tooth may go unrecognized on account of its distance from the gingival abscess.

3. The initial lesions of pyorrhea can only be determined by a study of the disease in its early stages; that is, at a time when they are not masked by other affections associated with it.

4. Precocious senile resorption of the alveolus is the initial lesion of pyorrhea; it is constant and precedes all the others.

5. This absorption has a general cause since it may be evolved independently of any local cause.

6. All local causes suggested as a cause of pyorrhea are only accessory causes since they may exist without pyorrhea, and the latter, especially in its early stages, may exist without them, which would be impossible if they played the part of primary causes.

7. Apart from all secondary local causes, pyorrhea is slowly progressive in proportion as the general causes which have brought about the senile prococious absorption of the alveolus continue.

8. Pyorrhea is first characterized by early absorption of the alveolus, but it would not pass on to the period of being fully established unless accessory local causes intervene to complicate it.

9. Prophylactic treatment of pyorrhea should include therefore the treatment of all accessory causes (rupture of articular equilibrium, infection, gingivitis, etc.). General treatment should be adopted in addition.

10. The normal functioning of teeth affected by pyorrhea is a factor favorable to their organic resistance; if it is destroyed it should always be restored.

11. Hygiene for patients suffering from pyorrhea should always be inspired by the essential principle that pyorrhea pockets only form round a tooth when some point of the gingival neck of the tooth is not kept sufficiently clean.

12. The dentist must, therefore, educate his patient whose co-operation is indispensable in the treatment and by regular examination treat the slightest complications of the disease.—*L'Odontologia,* June 30, 1919.

---

IODOFORM IN ROOT FILLINGS. Mix one part of iodoform with four parts of bismuth subnitrate and add enough formocresol to make a paste. To use add enough formocresol to make a thin paste of the quantity to be used to make a creamy consistence to facilitate application.—N. Rudin, in *Dental Digest.*

---

HANDLE FOR SMALL INVESTMENTS. Twist a piece of iron wire to form an oblong loop leaving the twisted ends as long as desired for a handle while soldering. Place the loop in the investment while it is soft in desired position.—Dr. Frahm, in *Pacific Gazette.*

